# Beyond CAR T Cells: Other Cell-Based Immunotherapeutic Strategies Against Cancer

**DOI:** 10.3389/fonc.2019.00196

**Published:** 2019-04-10

**Authors:** Shabnum Patel, Rachel A. Burga, Allison B. Powell, Elizabeth A. Chorvinsky, Nia Hoq, Sarah E. McCormack, Stacey N. Van Pelt, Patrick J. Hanley, Conrad Russell Y. Cruz

**Affiliations:** ^1^GW Cancer Center, The George Washington University, Washington, DC, United States; ^2^Center for Cancer and Immunology Research, Children's National Health System, Washington, DC, United States

**Keywords:** cell therapy, gene modified cells, immunotherapy, gamma delta T cells, NK cells, NKT cells, dendritic cells

## Abstract

**Background:** Chimeric antigen receptor (CAR)-modified T cells have successfully harnessed T cell immunity against malignancies, but they are by no means the only cell therapies in development for cancer.

**Main Text Summary:** Systemic immunity is thought to play a key role in combatting neoplastic disease; in this vein, genetic modifications meant to explore other components of T cell immunity are being evaluated. In addition, other immune cells—from both the innate and adaptive compartments—are in various stages of clinical application. In this review, we focus on these non-CAR T cell immunotherapeutic approaches for malignancy. The first section describes engineering T cells to express non-CAR constructs, and the second section describes other gene-modified cells used to target malignancy.

**Conclusions:** CAR T cell therapies have demonstrated the clinical benefits of harnessing our body's own defenses to combat tumor cells. Similar research is being conducted on lesser known modifications and gene-modified immune cells, which we highlight in this review.

## Introduction

Chimeric antigen receptors and engineered T cell receptors (based on previously identified high affinity T cell receptors) function by redirecting T cells to a predefined tumor-specific (or tumor-associated) target. Most of these modifications use retroviral or lentiviral vectors to integrate the construct, and most of the receptors feature a costimulatory signal—enhancing T cell activation following recognition of the target antigen. These modified T cells have collectively shown promising success rates, particularly against hematologic malignancies (1), with growing excitement for these novel treatments (2). Pioneering work at the NIH resulted in promising therapies for melanoma (3) and synovial sarcoma (4). Some of these therapies have been approved as licensed drugs.

CAR T cells targeting commonly overexpressed leukemia and lymphoma markers such as CD19 have shown promise in the prevention and treatment of malignancies such as Acute Lymphoblastic Leukemia (ALL), Chronic Lymphocytic Leukemia (CLL), Non-Hodgkin's lymphoma (NHL), Diffuse Large B cell lymphoma (DLBCL), and other B cell malignancies (5–8). These CD19-CAR Phase I and II trials have demonstrated safety and efficacy, with substantial partial and complete response rates (PR and CR, respectively). There are however, important concerns about toxicity—as resulting from on target off tumor effects, cytokine release syndromes, and neurotoxicity (9). Current CAR clinical trials are expanding to target other tumor-associated markers including GD2 (10), BCMA (11), CD20, CD30, CD33, CD7, HER2 (human epidermal growth factor receptor 2), and mesothelin (12–17). CAR T cells have been highlighted as Advance of the Year, by the American Society of Clinical Oncology in 2018 (18). A similar technology involves using high affinity T cell receptors (TCRs) and introducing these into cells (19). In the hopes of extending this success, other immune cell-based therapies are in current development.

The first group, non-CAR/non-TCR gene modified cell therapies for cancer, incorporates methods to overcome the barriers presented by cancer and the tumor microenvironment, as well as strategies for enhancing potency of T cell therapies. The second group focuses on immunotherapies generated from less frequently studied cell types including gamma-delta T cells, invariant natural killer T (iNKT) cells, natural killer (NK), and dendritic cells.

This review explores these lesser known cancer cell immunotherapy strategies, highlighting advances that have been made in recent preclinical and clinical efforts, and presents platforms for which they could demonstrate efficacy and may be critical for treating different cancer subtypes.

## NON-CAR/TCR Modification of T Cells

**Figure 1:**
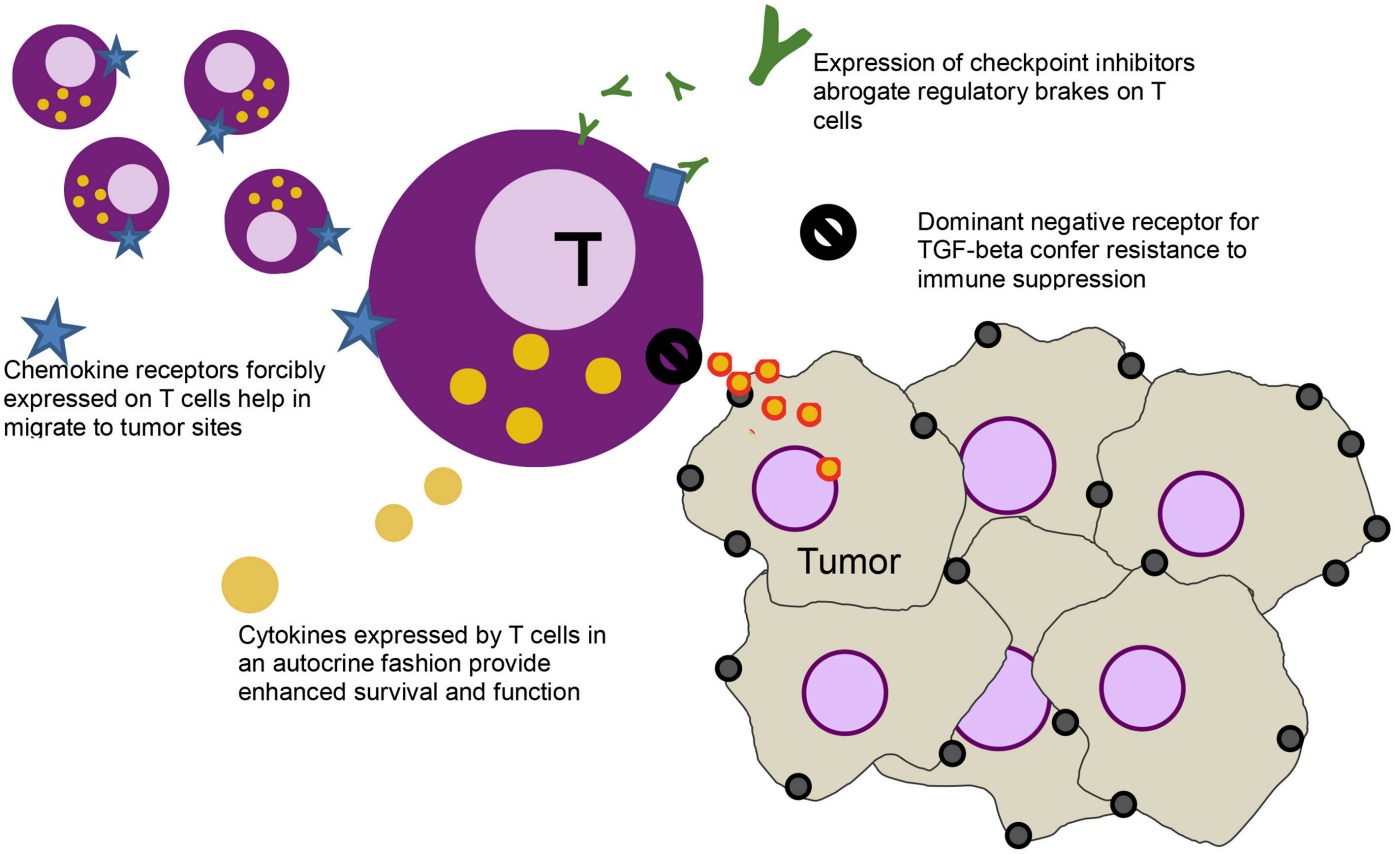
Schematic of a T cell (purple) and various modification - clockwise from left: forcibly expressed chemokine receptors (blue star) that help the cell migrate down the relevant chemokine gradient secreted by the tumor, secreted checkpoint inhibitors (green Ys) that bind to the checkpoint receptors on T cells (blue diamonds), abrogating their inhibitory function, a dominant negative receptor (black no sign) that helps block immune suppressive effects of cytokines like TGF-beta, and cytokines (yellow) that help stimulate the T cells in an autocrine function.

The ease by which T cells can be genetically modified has led to other gene modifications that aim to further enhance activity of T cells [a strategy that some groups have labeled as “armored” CARs (20), initially dubbed as “TRUCKS” (21)], including modifications to introduce dominant negative receptors, chemokine receptors, cytokines, cytokine receptors, and checkpoint inhibitors Figure 1.

### Dominant Negative Receptors

Translation of successful T cell therapies to solid tumors has been hampered by the immunosuppressive tumor microenvironment. Cancers secrete immunosuppressive cytokines which impair immune cell proliferation and function, and recruit regulatory T cells. These cytokines include TGFβ which inhibits the function of host immune cells (even those that successfully infiltrate the tumor), and induces epithelial-to-mesenchymal transition leading to cancer metastasis. Upregulation of TGFβ in the tumor microenvironment has been described in many aggressive malignancies including those of the brain, gastrointestinal tract, bone, breast, lung, and pancreas (22). TGFβ downregulates the secretion of critical Th1 cytokines, such as IFNγ, and impairs T cell and natural killer (NK) cell cytolytic activity and proliferation (23, 24).

A mutated form of the TGFβ receptor has previously been shown to exert a dominant-negative effect by abrogating the negative signaling cascade in cells that express this protein (25). This dominant negative receptor of the type II subunit (TGFβRII DNR) encompass the extracellular and transmembrane region of the endogenous cytokine receptor but exclude intracellular signaling domains, preventing downstream signaling when bound to ligand. Expression of this DNR has led to decrease in downstream signaling following TGFβ ligation—for example SMAD phosphorylation in the presence of TGFβ is abrogated by this receptor (26). T cells genetically engineered to express a TGFβRII dominant negative receptor (DNR) are resistant to the antiproliferative and anti-cytolytic effects of this cytokine (27). Genetically modified tumor antigen-associated T cells (in this case directed against Epstein-Barr virus antigens) expressing DNR show enhanced persistence and activity, resulting in superior antitumor activity (28). In this study, TGFβRII DNR restored proliferation of EBV-specific T cells in the presence of TGFβ, restored cytotoxicity against EBV-expressing lymphoblastoid cell lines, and demonstrated greater antitumor activity and migration *in vivo* (28). Other studies have also demonstrated the benefits of this DNR on the activity of T cells (see Table 1) (27, 29, 30, 32–34).

**Table 1:** Examples of preclinical research evaluating DNR-expressing T cells for the treatment of malignancies.

**Disease**	**Effector cell**	**Observed effects**	**References**
Prostate cancer	PSMA CAR T cells	Specific lysis of tumor, insensitivity to TGF-beta	(29)
EBV-positive lymphoma	EBV-specific T cells	Resist inhibitory effects of TGF-beta *in vitro* and *in vivo*, enhanced antitumor activity *in vivo*	(27)
Advanced prostate cancer	TCR-modified T cells	Complete and sustained tumor regression, enhanced cell survival, restored differentiation of prostate epithelium	(30)
Prostate cancer	PSMA CAR T cell	Increased proliferation, enhanced cytokine secretion, resistance to exhaustion, *in vivo* persistence, induction of tumor eradication in aggressive prostate cancer	(31)

A dose escalation study (using TGFβRII DNR antigen-specific T cells directed against EBV) of patients with EBV-positive lymphoma showed that these T cells were resistant to the inhibitory cytokine, with increased signals from peripheral blood, corresponding to increased frequencies of T cells. Persistence extended to more than 4 years, and four of seven evaluable patients had clinical responses (28). Other clinical trials incorporating TGFβRII DNR expressing cells have targeted a number of cancers including nasopharyngeal carcinoma (using antigen-specific T cells directed against EBV), metastatic melanoma (using tumor infiltrating lymphocytes TILs), EBV-positive Hodgkin disease and non-Hodgkin lymphoma using antigen-specific T cells directed against EBV), and HER2+ breast cancer (using chimeric antigen receptors directed against HER2) (see Table 2).

**Table 2:** Examples of clinical trials using various DNR-expressing T cells for the treatment of malignancies (35).

**Trial ID**	**Disease**	**Product**
NCT02065362	EBV-positive Nasopharyngeal carcinoma	TGFβ-resistant EBV-specific Cytotoxic T-lymphocytes +/– Cyclophosphamide and Fludarabine
NCT01955460	Metastatic melanoma	TGFβ-resistant T cells + Cytoxan, Fludarabine, Mesna, and Interleukin-2
NCT00368082	Relapsed EBV+ Lymphoma	TGFβ-resistant LMP-specific Cytotoxic T-lymphocytes (CTLs)
NCT00889954	HER2+ malignancies	TGFβ-resistant HER2/EBV-T cells

It is important to note that there may potentially be unintended consequences of conferring resistance to a regulatory cytokine: disruption of normal T cell homeostasis may result from expression of TGFβRII DNR. A study by Lucas et al. show that expression of the dominant negative receptor resulted in massive expansion of CD8 T cells in lymphoid organs (36). So far, no dysfunction has been observed in patients (28).

### Cytokine Receptors

Besides TGFβ, other negative/regulatory cytokines in the tumor environment limit T cell persistence and activity—these include IL10, IL13, and IL4. Another approach to reversing the immunosuppressive effects of these cytokines are chimeric cytokine receptors (CcR) (37). CcR's use the extracellular binding domain of an immunosuppressive cytokine bound to the intracellular signaling domain of an immune-activating cytokine to reverse its signaling effects. The first use of a chimeric IL4 cytokine receptor was described by Wilkie et al. where a fusion of IL4 receptor alpha ectodomain was fused to the subunit used by IL-2 and IL-15; this resulted in expansion and enhanced killing of MUC1 CAR T cells (38). In another study combining the extracellular domain of IL-4 cytokine receptor and the intracellular signaling domain of IL-7 cytokine receptor, CcRs restored the anti-tumor cytotoxicity of autologous T cells against EBV-transformed B cell tumors *in vivo* (37). In this study, CcR expression induced phosphorylation of STAT5 (part of the native signaling cascade in IL7 signaling) after ligation with tumor-secreted IL4, and restored T cell proliferation in the presence of the cytokine (37). This chimeric cytokine receptor also showed efficacy in a pancreatic cancer model: T cells modified to express a chimeric antigen receptor targeting prostate stem cell antigen (PSCA), found in pancreatic tumors, maintained their antitumor activity in an IL4-rich tumor microenvironment when they are co-transduced with the IL4/IL7 CcR (39). Another example uses a tumor-derived cytokine, CSF-1, to stimulate T cells by modifying these cells to express CSF-1R. Acquired responsiveness to CSF-1 allowed for improved chemotaxis and proliferation (40).

A simpler construct involves overexpression of a native cytokine receptor to allow for improved persistence following exogenous administration of the cytokine. One of the major challenges in T cell therapies is enhancing persistence of the cells *in vivo*. Previously, IL2 was administered to maintain T cell proliferation and activity (41), but IL2 is also associated with adverse effects (42)—limiting its applicability. IL7, on the other hand, provides the same effects without the unwanted toxicities. T cells, however, lose expression of the IL7 receptor after prolonged culture. In one study, genetic modification of EBV-specific CTLs to forcibly express IL-7 receptor α chain (IL-7Rα) led to restoration of CTL responsiveness to IL-7, and their antitumor activity sustained *in vivo* and *in vitro* without the unwanted toxicities related to IL-2 administration (43). In another study, cytokine feedback loops were used to improve efficacy of T cells by modifying these cells to express IL-7 and IL-21 (44).

### Cytokines

Select cytokines, like IL2, IL15, and IL12 perform stimulatory functions for T cells; in theory, autocrine secretion of these cytokines should help keep these cells persisting *in vivo*, even in the face of a hostile tumor environment (20).

In an example of this approach, CD19-CAR-specific T cells were modified to secrete IL15, and its anti-tumor efficacy evaluated using a xenogeneic model of lymphoma (45). In this study, IL-15 modified CD19 CAR T cells secreted IL15 following antigen stimulation, showed enhanced survival as a result of the transgenic cytokine, expanded better *in vivo*, and have better *in vivo* anti-tumor activity (45).

Other cell therapies incorporating cytokine secretion are listed in Table 3. One study, by Koneru et al. looked at MUC-16 specific T cells secreting IL12. Promising preclinical results (enhanced lysis of tumors and persistence *in vivo*) (49) led to its subsequent use in a phase I clinical trial for recurrent platinum-resistant ovarian cancer (50).

**Table 3:** Examples of preclinical research evaluating T cells expressing cytokines for the treatment of malignancies.

**Cytokine**	**Effector cell**	**Observed effects**	**References**
IL2	SWM specific T cell line 14.1	Autocrine growth without tumor formation, antigen specificity retained	(46)
IL12	Pmel-1 T cells	Enhanced lysis of established melanoma, no toxicities	(47)
IL15	Activated CD4+ and CD8+ lymphocytes	Continued proliferation after cytokine withdrawal, resistance to apoptosis	(48)

### Chemokine Receptors

One relatively underappreciated requirement for improving T cell therapies is successful migration to the site of disease (51). In the setting of malignancies, a possible avenue for improvement relies on the fact that tumors secrete chemokines that can potentially be harnessed to lead T cells to the tumor site. Chemokine receptors corresponding to the chemokines that are secreted by tumor cells have been introduced into T cells, which maximized efficacy of these therapies by improving localization (51).

The first chemokine receptor-engineered T cells redirected cells using CXCR2: these allowed cells to migrate toward the Gro-alpha chemokine gradient, and induced interferon gamma secretion from transduced T cells (52).

Moon et al. transduced the chemokine receptor CCR2b into mesoCAR T cells to treat tumors that express CCL2 and mesothelin (53). These modified T cells improved tumor localization, a limitation of CAR-based approaches, and showed enhanced anti-tumor activity. Craddock et al showed that in neuroblastoma cell lines derived from six patients, modified activated T cells showed a 60% increase in the expression of CCR2b and co-expressed CCR2b and GD2-CAR showed a 10-fold improvement in migration to the tumor site compared to CCR2 negative activated T cells (54). Using the transgenic adenocarcinoma of mouse prostate (TRAMP) model, Garetto et al. (55) showed that expressing chemokine receptors on T cells tailored for chemokines that are strongly secreted in the tumor milieu can be used to improve targeting of T cells.

Additional examples are listed in Table 4.

**Table 4:** Examples of preclinical research evaluating T cells expressing chemokine receptors for the treatment of malignancies.

**Chemokine receptor**	**Effector cell**	**Observed effects**	**References**
CX3CR1	Activated T cells	Enhanced lymphocyte migration and tumor trafficking, significant inhibition of tumor growth	(56)
CCR2	WT1-TCR-modified T cells	CCL2-tropic tumor trafficking, cytocidal reactivity against WT1-expressing cells, augmentation of TCR signaling	(57)
CCR4	CD30 CAR T cells	Enhanced migration to Hodgkin lymphoma cells, sustained cytotoxic function and cytokine secretion *in vitro*, enhanced tumor control *in vivo*	(51)

### Checkpoint Inhibitors

In addition to T cell therapies, the introduction of checkpoint inhibitors has been responsible for the interest in immunotherapies. These molecules, typically antibodies directed against checkpoint receptors expressed on T cells, inhibit negative regulation of these cells—removing the “brakes” to their activity. Combinations of T cell therapies and checkpoint inhibitors are therefore particularly attractive. Administration of a PD-1 blocking antibody enhanced CAR T cell function against established tumors (58).

One way to coordinate spatiotemporal activity of these therapeutics is to have T cells directly secrete these inhibitors. One group engineered CD19 CAR T cells to secrete single chain variable fragments targeting PD1. T cells were shown to secrete functional anti-PD1 scFv (~600 ng/mL), capable of reversing PD1/PDL1 interactions and their negative effects on T cell function. This allowed for enhanced T cell expansion and effector function *in vitro* and *in vivo* (59). Another group also modified various CAR T cells to secrete PD1 blocking scFV and showed improved antitumor activity, as well as bystander tumor-specific T cell activity, in syngeneic and xenogeneic murine models of tumors expressing PDL1 (60). Other groups knocked down expression of PD-1 (61) or components of PD-1 signaling, to improve function of adoptively transferred cells (62).

## Other Immune Cells

Although the specific, direct actions of gene-modified T cells are mostly responsible for the promising clinical results—indirect effects mediated through other immune cells also contributed to efficacy. In addition, there is an increasing body of evidence that suggests engagement of multiple arms of immunity are key toward longer lasting resolution of tumor.

**Figure 2:**
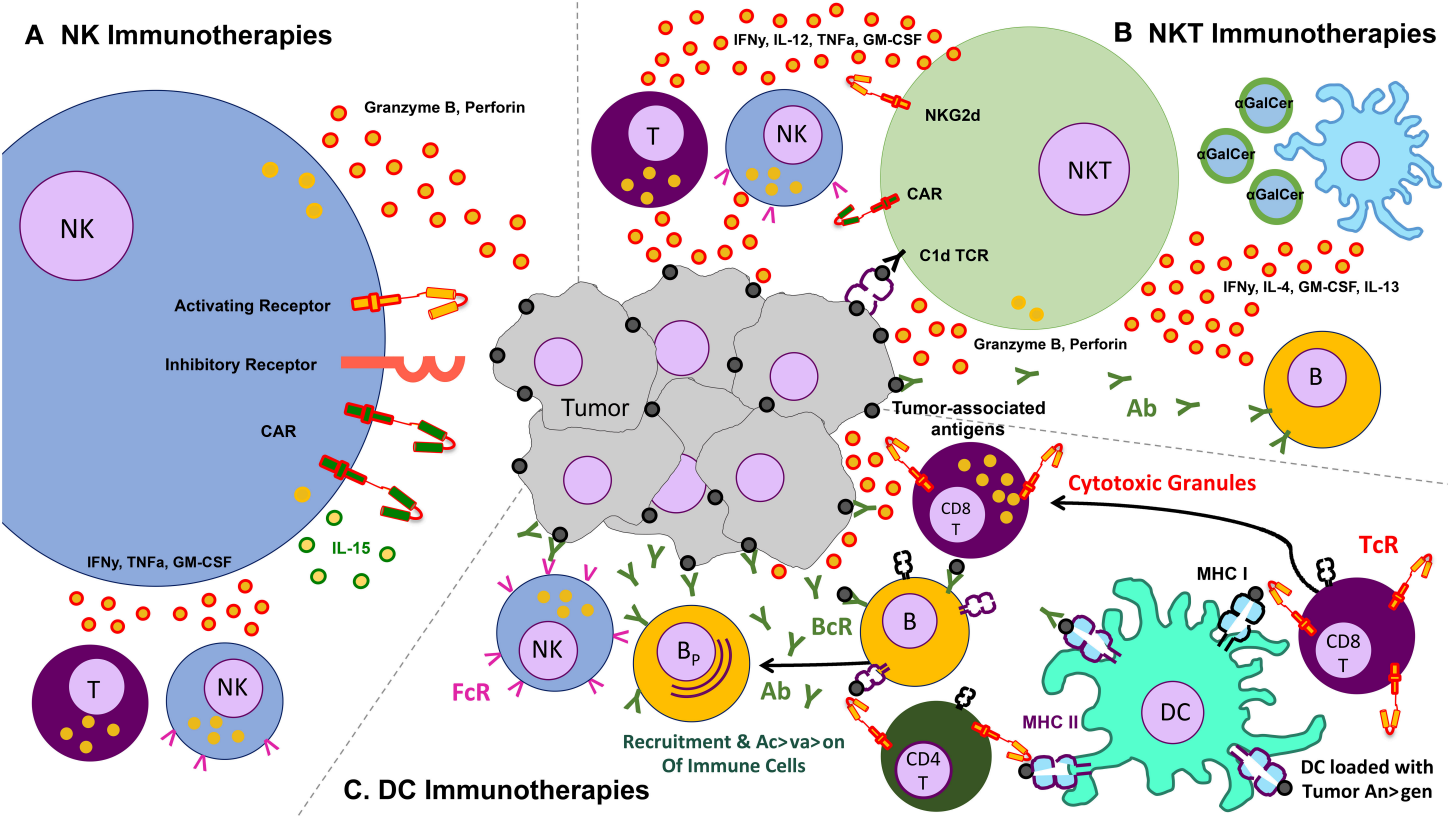
Schematic of various other cells and their effects on the tumor – clockwise from left: **(A)** natural killer (NK) cells, which lyse tumors without the need for identifying a known antigen, working through a balance of inhibitory and activating receptors (and also amenable to transduction with chimeric antigen receptors), as well as secrete cytokines that activate other components of the immune response, **(B)** natural killer T (NKT cells, which recognize lipid antigens and also help orchestrate the immune response, **(C)** dendritic cells (DC) which present antigen to T cells and help jump start immune responses like a vaccine.

The use of other immune cells as immunotherapies for cancer is therefore a necessary adjunct to the existing T cell therapies. Some of the more commonly studied cells include gamma-delta (γδ) T cells, invariant natural killer T (iNKT) cells, natural killer (NK), and dendritic cells. We limit this section to these endogenously occurring cells, though acknowledge that other cells that can be expanded *ex vivo*—e.g., cytokine induced killer cells (CIK)—may form a potentially efficacious immune therapeutic (Figure 2).

### Gamma-Delta T Cells

γδ T cells are a small subset of cells, whose functions make them attractive candidates for potential immunotherapies. γδ T cells have many innate like properties, and similar to other innate cells, such as NKs, γδ T cells express NK receptor NKG2D and show cytotoxicity to tumor cells (63). Two groups of γδ T cells are recognized, based on the TCR V delta usage: V delta 1 cells are located in mucosal tissue, and V delta 2 cells are located in the peripheral blood (64). V delta 2 cells are a source of proinflammatory cytokines once activated, including TNF- α and IFN-γ (64). The mechanisms by which γδ T cells recognize cancer are not fully understood. They can recognize tumor antigens via their TCRs and NK receptors, but it is unclear what specific antigens they respond to (65). γδ2 T cells typically recognize pyrophospate antigens produced by bacteria, while γδ1 T cells recognize MHC class I related molecules like MICA/MICB (64). In the cancer setting, it is thought that γδ T cells recognize stress induced self-like antigens, typically expressed by malignant cells and found to infiltrate tumors in some cases (66). These cells appear to mediate a graft vs. tumor response without eliciting GVHD (67).

In pre-clinical studies, γδ T cells have been expanded and have demonstrated cytotoxicity to a variety of tumor cell lines derived from lung carcinoma, liver cancer, and breast cancer, in an MHC-unrestricted manner (66). Deniger et al. demonstrated that they were able to see a 107-fold increase in γδ T cell numbers, despite a small starting population, suggesting it is possible to expand to clinically relevant numbers (68). Another study by Liu et al. show that γδ T cells have the ability to recognize and kill some forms of prostate cancer *in vitro* via innate mechanisms (69). In other preclinical studies, it was demonstrated that γδ T cells could be transduced to generate CAR-T cell products that maintained their natural tumor infiltration and killing abilities (70).

Some clinical trials using these cells are already underway In a Phase I study, autologous γδ T cells were infused in combination with IL-2 into 10 patients with metastatic renal cell carcinoma (mRCC) (71). This trial demonstrated safety, as infusions were tolerated with few serious adverse events related to the immunotherapy, with six patients showing stable disease. In another study, patients with hepatocellular carcinoma were given an injection of γδ T cells (NCT00562666).

Although γδ T cells have been well-tolerated in cancer patients, they are limited by difficulties in their isolation (65), and some questions surround their potential tumor-promoting effects (effects on angiogenesis and secretion of IL-17) (72, 73).

### Natural Killer Cells

Natural killer cells were initially identified for their ability to target and kill tumor cells (74). They exhibit cytolytic function through the release of perforin and granzyme B as well as through FasL-TRAIL-mediated pathways, and NK cell activity is governed by a balance of signals from both activating and inhibitory receptors (75–78). NK cells are an possible option for adoptive immunotherapy because they do not require prior antigen exposure to elicit cytotoxicity. In addition, NK cells have limited persistence *in vivo*, a feature that appeals to clinicians and scientists alike. There is preclinical and clinical evidence that NK cells do not cause graft vs. host disease (GVHD) (79–83) or result in systemic toxicities associated with “cytokine storms” seen in T cell therapies (84–86). Similar to other new immunotherapies, an initial roadblock to the clinical use of NK cells was the inability to expand NK cells to clinically relevant numbers.

An additional challenge facing NK cells for adoptive therapy is the immunosuppressive tumor microenvironment, which directly nullifies the cytotoxicity of NK cells (87). Specifically, there is an abundance of immunosuppressive cell types such as myeloid-derived suppressor cells (MDSC) (88–90), tumor-associate macrophages (TAM) (91), and regulatory T cells (Treg) (26, 92–95), as well as cytokines such as transforming growth factor beta (TGFβ) and indoleamine 2,3 dioxygenase (IDO) (26, 96), that have been shown to interact with NK cells and cause phenotypic and functional dysfunction. Many groups have performed preclinical work in order to exploit the anti-tumor, cytotoxic, capabilities of NK cells, while addressing the challenges faced by adoptive cell therapy. For instance, Mentlik et al. focused on these combining NK cell therapy with monoclonal antibodies, boosting NK cell's ability to conduct ADCC (97); these combination therapies with antibodies or cytokines are the focus of other preclinical efforts (98).

Extensive effort has been put into generating and characterizing NK cells for adoptive cell therapy from both primary donor and immortalized NK line donor sources, with mixed results (Table 5). As with T cells, there is tremendous appeal for equipping cytotoxic cells with the ability to specifically recognize and kill a given tumor target—as such, there have been multiple attempts at generating CAR-NKs, that retain their cytotoxicity but are instead directed toward a specific antigen (122). CAR-NKs targeting B cell malignancies have demonstrated impressive *in vivo* cytolytic efficacy (123–126), and represent a promising transition of the technology to the clinic. Other modifications have been incorporated in NK cells—in one such study, cord blood NK cells engineered to express IL15 and a CD19 CAR showed marked increase in survival in a xenograft lymphoma model (127).

**Table 5:** NK cell production methods.

**Donor source**	**Cell population**	**NK purity**	**Cell expansion method**	**References**
Umbilical cord blood	CD34+ hematopoietic stem cells	>90%	Differentiation and expansion in bioreactor with GM-CSF, G-CSF, IL-6, IL-7, SCF, IL-15, and IL-2	(99, 100)
Umbilical cord blood	CD34+ hematopoietic stem cells		Differentiated and cultured with IL-15 and MS-5 or OP9 stromal feeder cells	(101)
Umbilical cord blood	CD34+ hematopoietic stem cells	>90%	Cultured with StemRegenin-1,IL-7, SCF, IL-15, and IL-12	(102)
Umbilical cord blood	CD34+ hematopoietic stem cells	>80%	Cultured with high dose cytokine cocktail and OP9 or M2-10B4 feeder cells	(103)
Umbilical cord blood	Total mononuclear cells	90%	Cultured with K562 Clone 9.mbIL21 feeder cells, IL-2, IL-12, IL-15, and IL-18	(104)
Umbilical cord blood	CD56+ NK cells		Cultured with irradiated feeder cells (K562-C9), IL-15, and IL-2	(26)
Peripheral blood	Total PBMC	>80%	Cultured with irradiated feeder cells (K562-C9)	(105)
Peripheral blood	Total PBMC	>55%	Cultured with IL-2 and OKT3	(106–109)
Peripheral blood	Total PBMC	90%	Cultured with OK432, IL-2, and modified FN-CH296 induced T cells	(110)
Peripheral blood	Total PBMC	>80%	Cultured with low dose IL-2 and RPMI 8866 feeder cells	(111, 112)
Peripheral blood	Total PBMC	>90%	Cultured with IL-2 and LAZ388 feeder cells	(113)
Peripheral blood	CD56+ NK cells		Cultured with IL-15 and IL-2	(114)
Peripheral blood	CD5 and CD8 depleted PBMC	>88%	Cultured with high dose IL-2	(115, 116)
NK-92 Cell line	Immortalized NK cell line	100%	Cultured with IL-2	(117, 118)
KHYG-1 Cell line	Immortalized NK cell line	100%	Cultured with IL-2	(119)
NK-YS Cell line	Immortalized NK cell line	100%	Cultured with IL-2 and SPY3-2 feeder cells	(120)
haNK Cell Line	Immortalized NK cell line (based on NK-92)	100%	Cultured with IL-2	(121)

To date, three trials with genetically modified primary NK cells, and are currently active (NCT03056339, NCT00995137, NCT01974479). Existing clinical CAR-NK therapies borrow directly from the manufacturing schemes in the CAR-T cell field. One new approach involves substitution of the CD3ζ domain, which initiates TCR-based activation in T cells, with an intracellular domain that is specifically involved in NK cell activation. Indeed, NK-specific activation domains DNAX Activating Protein 10 (DAP10) and 12 (DAP12) have been introduced as the intracellular component in a CAR-NK in preclinical work, and promising results have demonstrated enhanced NK activation and function with this modification (128, 129).

In addition to the abovementioned CAR-NK clinical efforts, multiple clinical trials are underway using infusions of either autologous or allogenic NKs, with more promising results occurring in patients treated with allogenic NKs [reviewed in (130, 131)]. A study by Burns et al. using *ex vivo* activated NKs for treating patients with Hodgkin's and renal cell carcinoma was unable to demonstrate clinical efficacy (132), perhaps due to the autologous donor source. Furthering this claim were the results from multiple groups that demonstrated enhanced NK cell cytotoxicity occurring in patients if there was a killer immunoglobulin receptor-human leukocyte antigen (KIR-HLA) mismatch between donor and recipient cells (83, 93). One of the outstanding challenges for the use of adoptive NK cell therapy pertains to the cells' innate sensitivity to the freeze-thaw process. Indeed, preclinical reports have demonstrated impaired viability and cytotoxicity following cryopreservation (133, 134).

In addition to improving the manufacture end of NK cells therapies, developments are underway that aim to enhance the functionality and persistence of these therapies. For instance, focus for NK cell as well as other cell therapies has shifted toward modulating the suppressive tumor microenvironment concurrently with cell therapy in order to enhance efficacy (26). Moreover, a class of immunomodulatory drugs, such as thalidomide, have been found to modify the NK cells in the tumor environment by upregulating surface expression of TRAIL, which may increase NK-mediated apoptosis of target tumor cells (135–137). Miller et al. are developing bi-specific killer engagers (BiKEs) and tri-specific killer engagers (TriKEs) that can address many of the challenges facing NK cell therapy all in one construct (138–141). They have developed a platform by which NK cells are rendered specific for a given target antigen, while simultaneously increasing NK cell potency and persistence by incorporating CD16 single chain variable fragment (to increase ADCC-associated signaling) and an IL15 moiety (to increase NK activation and thus persistence). These findings that BiKE and TriKE-modified NK cells delivered potent anti-tumor responses in the setting of AML, ALL, and CLL, as well as the extensive number of ongoing clinical trials are only one example of how the field of immunotherapy is rapidly expanding to include a variety of non-T cell-based immunotherapies.

### Natural Killer T Cells

NKTs represent an important link between the innate and adaptive immune system, as they can be activated by both antigen dependent and antigen-independent mechanisms. Divided into invariant (iNKT) or diverse (dNKT) subsets, they have a highly restricted TCR repertoire, only recognizing antigen in the context of the MHC class I-like CD1d molecule (142), and are uniquely classified by their ability to rapidly produce regulatory cytokines such as IFNγ, IL4, IL10, IL13, IL-17, GM-CSF, and TNFα in large quantities (143). These characteristics together contribute to the appeal of this cell subset as a form of immunotherapy. Although populations of iNKT cells isolated from cancer patients have been found to be decreased in quantity and defective (144–146), many groups have shown that this impaired phenotype is in fact reversible *ex vivo* (147–150). Additionally, preclinical studies have supported the promise of NKT therapy as a multimodal platform—the glycolipid alpha-galactosylceramide (αGalCer) can reactivate impaired NKTs *ex vivo* to result in restored cytokine production and anti-tumor responses (151–154). Further, inhibition of tumor progression has been demonstrated in models of colon carcinoma, lymphomas, sarcoma, melanoma, prostate cancer, and lung cancer, leading to resurgence of optimism in iNKT cells as agents of immunotherapy.

NKTs are of particular interest as a possible cell for CAR modification for two main reasons: first, because clinical data has indicated better outcomes occurring in patients with higher NKT cell tumor infiltrate (155, 156), and second because the CD1d restricted nature of NKT antigen recognition is able to limit the potential off-target toxicity and increase potential applicability in both the autologous and allogeneic setting (157). Because NKTs secrete a wide range of regulatory cytokines, they are able to both activate antigen presenting cells such as dendritic cells as well as cytotoxic cells such as CD8+ T cells and NK cells—further increasing their value as an agent of immunotherapy (Figure 2) (158–163). Heczey et al. generated CAR-modified NKT cells to target neuroblastoma (aGD2 CAR) and lymphoma (aCD19 CAR), with marked success. They found that their CAR NKT cells had highly potent and selective cytotoxic activity against tumor target antigen-expressing cells, and were able to efficiently proliferate and produce large amounts of cytokines in the tumor environment, thus mediating their efficacy (164). Rotolo et al. generated CAR CD19-modified NKT cells to better target CD19-expressing lymphomas that also express CD1d, the ligand for NKT (165).

Many attempts have been made to directly target and restore function to patients' endogenous NKT cells, and current trials are summarized in Table 6. This avenue has focused on the infusion of NKT cell activating or stimulating agents, largely αGalCer (166), or by combining these agents with APCs such as dendritic cells to enhance immune activation at the suppressed tumor site (167–173). Dendritic cells can be pulsed with glycolipid and reintroduced into patients, a strategy regularly used in vaccine development, which has been proven to induce activation and restore function to endogenous NKT cells in a range of cancer types (167, 174–176).

**Table 6:** Examples of clinical trials with iNKT cells (35).

**Trial ID**	**Disease**	**Product**
NCT00003985	Solid Tumors	KRN7000 (alpha gal-cer)
NCT00698776	Myeloma	Combination of Lenalidomide and dendritic cells loaded with KRN7000 (alpha gal-cer)
NCT03093688	Advanced Solid Tumors	Infusion of iNKT cells and CD8+T cells
NCT02562963	Non-small cell lung cancer, gastric cancer, hepatocellular carcinoma, colorectal cancer	NKT cells expanded from PBMC
NCT01801852	Breast Cancer, Glioma, Hepatocellular Cancer, Squamous Cell Lung Cancer, Pancreatic Cancer, Colon Cancer, Prostate Cancer	Autologous NKT cells
NCT03198923	Non-small cell lung cancer	NK and NKT cells
NCT03294954	Neuroblastoma	NKT cells genetically modified to express a GD2-CAR
NCT00909558	Breast Cancer, Glioma, Hepatocellular Cancer, Squamous Cell Lung Cancer, pancreatic Cancer, Colon Cancer, Prostate Cancer	Autologous NK or NKT cells
NCT01235845	Malignant Glioma	DC-activated NKT cells and DCs

The largest challenge facing the advancement of CAR and non-CAR NKT cell therapies is that of persistence; tumor progression negatively correlates with NKT cell functionality. Attempts to subvert this impairment in NKT function include efforts where autologous NKTs are expanded *ex vivo* with αGalCer prior to reinfusion, as previously described (177). Attempts have been made to classify the phenotype of NKT cells during tumor progression, and CD62L has been identified as a potential indicator of NKT cells most likely to demonstrate enhanced anti-tumor activity (178). Moreover, new approaches to drug or glycolipid delivery systems are currently in development, which aim to package agents causing activation of NKTs in enhanced nanoparticle-based constructs. Examples of this novel immunotherapy “associated agent,” such as αGalCer packaged into microspheres or liposomes, have demonstrated enhanced NKT functional responses as compared to the agent alone (179–182). These modifications to CAR and non-CAR NKTs speak to the tremendous promise of generating enhanced clinical NKT therapies.

### Dendritic Cells

Dendritic cells (DCs), one of the professional antigen-presenting cells of the immune system, efficiently process antigens for presentation to T cells in order to activate the adaptive immune system (183). DCs naturally play a role in the control of immune responses and immune tolerance, both critical in anti-tumor immunity (183–185). Pre-clinical *in vivo* mouse models of cancer have demonstrated that DCs have the ability to home to tumor sites and capture tumor-associated antigens for processing. These DCs subsequently travel to nearby lymph nodes, where they present tumor antigens to T cells, generating tumor-specific T cells that can lead to clearance or tumor rejection (184, 185). Furthermore, DCs have the unique role of interacting with several subsets of the immune system, including both CD4 and CD8 T cell subsets in lymph nodes, resulting in downstream B cell activation into antibody-secreting cells, as well as activation of NKs and phagocytes. For example, in a murine model of melanoma, it was demonstrated that DCs interact with both cytotoxic T cells and NK cells to mediate tumor elimination (186, 187). However, NK depletion resulted in no tumor elimination, emphasizing the importance of DC-NK interactions in anti-tumor immunity (186, 187). This ability to interact with and regulate multiple immune cells make DCs an interesting candidate cell subset to be used in immunotherapy trials.

Due to their natural role in antigen processing and presentation, dendritic cells have been used in multiple Phase III clinical trials as an adjuvant or therapeutic vaccine for certain cancers including metastatic melanoma (NCT01875653), prostate cancer (below), renal cell carcinoma (NCT01582672), and glioblastoma multiforme (NCT00045968) (188, 189) (Table 7). The main objective of these studies was to deliver tumor antigens via DCs to stimulate and activate anti-tumor antigen-specific T cells, which subsequently eliminate cancerous cells and provide immunological memory to prevent tumor relapse. Furthermore, it has been demonstrated that the induction of anti-tumor T cell responses from DC-immunotherapies concurrently enhances natural killer immunity (187), underscoring the importance of DCs in regulating multiple immune cell subsets (Figure 2).

**Table 7:** Examples of clinical trials with dendritic cells (35).

**Trial ID**	**Disease**	**Product**
NCT01875653	Metastatic melanoma	Autologous dendritic cells loaded with irradiated autologous tumor cells in GM-CSF
NCT00005947, NCT01133704	Metastatic prostate cancer that has not responded to hormone therapy	Sipuleucel-T
NCT00065442	Metastatic prostate cancer that has not responded to hormone therapy	Sipuleucel-T
NCT00779402	Early stage, non-metastatic prostate cancer	Sipuleucel-T
NCT01582672	Renal cell carcinoma	AGS-003 (Autologous dendritic cell product)
NCT00045968	Glioblastoma multiforme	DCVax-L
NCT01067287	Multiple myeloma	Pidilizumab (CT-011) + Dendritic cell-myeloma fusion vaccine
NCT01096602	Acute myelogenous leukemia (AML)	Dendritic cell-AML fusion vaccine
NCT01441765	Renal cell carcinoma	Pidilizumab (CT-011) + Dendritic cell-renal cell carcinoma fusion vaccine

One of the significant advantages with DC-based immunotherapies is the demonstration of safety across multiple clinical trials (188–191), with promising efficacy shown in certain cancer settings. For example, in a Phase 3 IMPACT study for prostate cancer, DC-based therapy, sipuleucel-T, demonstrated significantly better survival by 4 months, for patients with metastatic hormone-resistant prostate cancer compared to the placebo group (191). Multiple Phase 3 prostate cancer studies (NCT00005947, NCT00065442, NCT00779402, NCT01133704) with DC-immunotherapy sipuleucel-T have shown induction of antigen-specific immune responses correlate with better survival in patients (190–194) (Table 7). Because of observed improvements in survival, sipuleucel-T was FDA approved in 2010. It is interesting to note that this coincides with <5% patients achieving an objective response, or tumor reduction over time.

Current clinical strategies are looking to optimize DC immunotherapy through combinations with other agents, in an effort to improve tumor burden. For example, the immunosuppressive tumor environment may prevent DCs from effectively activating cytotoxic T cells and NK cells to eliminate the tumor. Consequently, immune checkpoint inhibitors such as pidilizumab, are currently being explored in combination with DC immunotherapies (NCT01067287, NCT01096602, NCT01441765) for multiple myeloma, acute myelogenous leukemia (AML), and renal cell carcinoma, in an effort to enhance activation of tumor-specific cytotoxic T cells by DCs (195, 196) (Table 7). Ultimately, DC immunotherapies have shown promise in certain cancer settings, and have the advantage of interacting with numerous immune cell subsets to mediate anti-tumor immunity. The efficacy of these DC immunotherapies may be improved upon through combination strategies with other agents and the targeting of immunosuppressive barriers to tumor eradication.

## Conclusions

CAR T cell therapies have demonstrated the clinical benefits of harnessing our body's own defenses to combat tumor cells. Similar research is being conducted on lesser known modifications and gene-modified immune cells. Promising preclinical and clinical results point to a likely establishment of these therapeutics as another treatment modality against cancer. Because the field is a recent one, it is necessarily disjointed: different groups focus on their preferred immune effector and seldom compare efficacy with others, much less look at potential combinations. By presenting this review, the authors hope that researchers become more familiar with what is out there—and hope that more efforts at head-to-head comparisons between therapies and combination therapies (which is how the immune system is supposed to act) be explored.

## Author Contributions

SP and CC conceptualized the review and made edits to the manuscript. SP, RB, EC, AP, SV, NH, SM, and CC wrote the body of the text. PH and CC made final edits to the manuscript.

### Conflict of Interest Statement

PH and CC are co-founders of Mana Therapeutics, a biotech startup focusing on cell therapies. PH is a member of the board, and CC is a member of the scientific advisory board. The remaining authors declare that the research was conducted in the absence of any commercial or financial relationships that could be construed as a potential conflict of interest.

## Abbreviations

ALL: Acute lymphocytic leukemia
AML: Acute myeloid leukemia
BCR: B cell Receptor
BMT: Bone Marrow Transplant
CAR: Chimeric Antigen Receptors
CLL: Chronic lymphocytic leukemia
CRISPR: Clustered Regularly Interspaced Short Palindromic Repeats
DC: Dendritic Cell
DLBCL: Diffuse Large B Cell Lymphoma
DNR: Double Negative Receptor
ESC: Embryonic Stem Cell
FcR: Fc Receptor
HLA: Human Leukocyte Antigen
HSCT: Hematopoietic Stem Cell Transplant
iPSC: Induced Pluripotent Stem Cell
MHC: Major Histocompatability Complex
mRCC: metastatic renal cell carcinoma
NHL: Non-Hodgkin's Lymphoma
NK: Natural Killer Cell
NKT: Natural Killer T Cell
TALEN: Transcription Activator-Like Effector Nuclease
TCR: T cell Receptor
ZFN: Zinc Finger Nuclease.
